# Pre-Hospital Delay in Acute Ischemic Stroke Care: Current Findings and Future Perspectives in a Tertiary Stroke Center from Romania—A Cross-Sectional Study

**DOI:** 10.3390/medicina58081003

**Published:** 2022-07-27

**Authors:** Elena Oana Terecoasă, Răzvan Alexandru Radu, Anca Negrilă, Iulian Enache, Bogdan Cășaru, Cristina Tiu

**Affiliations:** 1 Department of Neurology, University Emergency Hospital Bucharest, 050098 Bucharest, Romania; oana_ter@yahoo.com (E.O.T.); negrila.anca09@gmail.com (A.N.); iulian93@hotmail.com (I.E.); cbogdan185@gmail.com (B.C.); cristinatiu@yahoo.com (C.T.); 2 Department of Clinical Neurosciences, “Carol Davila” University of Medicine and Pharmacy Bucharest, 020021 Bucharest, Romania

**Keywords:** pre-hospital delay, onset-to-door time, ischemic stroke, reperfusion therapy, intravenous thrombolysis

## Abstract

*Background and objectives*: The time interval between stroke onset and hospital arrival is a major barrier for reperfusion therapies in acute ischemic stroke and usually accounts for most of the onset-to-treatment delay. The present study aimed to analyze the pre-hospital delays for patients with acute ischemic stroke admitted to a tertiary stroke center in Romania and to identify the factors associated with a late hospital arrival. *Material and methods*: The study population consisted of 770 patients hospitalized with the diagnosis of acute ischemic stroke in the University Emergency Hospital Bucharest during a 6-month period, between 1 January and 30 June 2018. Data regarding pre-hospital delays were prospectively collected and analyzed together with the demographic and clinical characteristics of the patients. *Results*: In total, 31.6% of patients arrived at the hospital within 4.5 h from stroke onset and 4.4% in time intervals between 4.5 and 6 h from the onset, and 28.7% of the patients reached the hospital more than 24 h after onset of symptoms. Transport to hospital by own means was the only factor positively associated with arrival to hospital > 4.5 h from stroke onset and more than doubled the odds of late arrival. Factors negatively associated with hospital arrival > 4.5 h after stroke onset were prior diagnosis of atrial fibrillation, initial National Institute of Health Stroke Scale (NIHSS) score ≥ 16 points, presence of hemianopsia, facial palsy and sensory disturbance. Factors increasing the odds of hospital arrival after 24 h from stroke onset were living alone and living in rural areas. *Conclusions*: Almost one in three ischemic stroke patients presenting to our center reaches hospital more than 24 h after onset of symptoms. These findings highlight the need for urgent measures to improve not only stroke awareness but also pre-hospital protocols in order to provide timely and appropriate care for our stroke patients.

## 1. Introduction

The global burden of stroke is overwhelming. In 2016, an estimated 5.5 million deaths (10.1% of all deaths) were caused by stroke, and there were 13.7 million new stroke cases worldwide with an overall cost of 116 million disability-adjusted life years [1,2]. Almost 70% of strokes are ischemic [3] and may have a multitude of etiologies. However, acute ischemic stroke management is not primarily based on its cause but on a thorough understanding of the cascade of pathophysiological mechanisms unfolding during the acute phase. The introduction of the concept of “ischemic penumbra” in the 1970s [4] was the cornerstone for the huge change in acute ischemic stroke care that we have witnessed over the past two decades. Intravenous thrombolysis (IVT) and endovascular treatment (EVT), both targeting rapid restauration of the blood flow and salvage of the penumbral tissue, have been shown to improve stroke outcome when administered in a specific time interval [5,6]. However, due to the strict criteria of eligibility and the costs associated with establishing the needed infrastructure, these therapies are restricted to only a small percentage of patients [7]. A key element for providing IVT or EVT in a limited time window to a large population of stroke patients is the implementation of well-organized stroke networks that require substantial financial and human resources. However, these networks will not have the expected effect without continuous efforts of the public health leaders and medical professionals aimed to increase early stroke symptom recognition and to improve timely access to emergency care. 

Late patient presentation to hospital is perhaps the most important barrier to the widespread utilization of IVT in stroke care. Over the 25 years that have passed since the completion of the NINDS trial, many studies have assessed the underlying reasons for not administering recombinant tissue plasminogen activator (rtPA) to acute ischemic stroke patients [8]. With almost no exception, these studies have shown that in hospitals with active protocols for IVT, the main reason for not administering rtPA to stroke patients was their late arrival at the hospital [9]. A review including several studies from different countries and time periods showed that only 21% to 40% of all ischemic stroke patients arrive at hospitals within 3 h from stroke onset and another 5–13% within 3–6 h [10]. Despite this constant finding that delayed hospital arrival is a major factor contributing to low IVT rate, the analysis of stroke onset to hospital arrival time intervals reported in the studies performed between 2008 and 2016 revealed that the percentage of stroke patients arriving at hospital in due time for IVT had shown only little improvement over the years [9].

To our knowledge, no data about pre-hospital delays for acute stroke patients are officially available for Romania, and data from other Eastern European countries are scarce. The aim of our study was to determine the percentage of stroke patients that reach the hospital in the early (4.5 h), late (4.5–24 h) and very late time window (>24 h) after the onset of symptoms and to describe the factors that are associated with late and very late hospital arrival in our catchment area.

## 2. Materials and Methods

We performed a cross-sectional study conducted during a six-month period, between 1 January 2018 and 30 June 2018, in the University Emergency Hospital Bucharest, Romania. No public campaigns regarding stroke awareness and no educational stroke programs for the personnel of the Emergency Medical Services (EMS) were taking place before or during the study.

All patients hospitalized in the Department of Neurology with the diagnosis of acute ischemic stroke between 1 January and 30 June 2018 were screened for inclusion in the study. Patients with in-hospital strokes, those with stroke mimics and those for which data regarding past medical history, prior treatment, moment of stroke onset or other important details needed for the study purpose could not be obtained were excluded. A questionnaire regarding the time when the patient was last seen without symptoms and hospital arrival time was completed by the neurologist on duty for every patient with ischemic stroke within 48 h from hospital admission by interviewing the patient and/or the family members. For patients without cognitive impairment, aphasia or confusion, this information was obtained from the patient. If the patient had aphasia, confusion or cognitive impairment, details about the time of stroke onset were obtained from family, caregivers or stroke witnesses. Data regarding socio-demographic characteristics, past medical history, modified Rankin score (mRS) before stroke, vascular risk factors, prior treatment, clinical presentation, National Institute of Health Stroke Scale (NIHSS) score at admission, vital parameters at admission, laboratory and imaging findings were collected in a prespecified manner from the medical records. For all patients included in the study, data about reperfusion therapy received (IVT, EVT or none) was recorded. The diagnosis of acute ischemic stroke was established by the neurologist on duty on the basis of clinical signs and a brain computed tomography (CT). Stroke onset was defined as the moment when the patient was last known to be without symptoms. If the patient awoke with symptoms, stroke onset was defined as the time when the patient went to sleep or was the last known to awake without symptoms. 

Statistical analysis was performed using NCSS Statistical Software version 20.0.3 (NCSS, LLC. Kaysville, UT, USA). The Kolmogorov–Smirnov test and the aspect of the histograms were used to assess the data distribution. Continuous variables are presented as mean values and standard deviation for normally distributed data and median values and 25–75 interquartile range for non-parametric data. Categorical variables are presented as absolute values and percentages. The pre-hospital time intervals were dichotomized according to different cut-off values in order to explore factors related to longer time intervals. Univariate analysis was first performed using Mann–Whitney U test for continuous variables and Pearson’s χ2 test or Fisher’s exact test (as appropriate) for categorical variables. Explanatory variables with a significance level of *p* < 0.1 were entered into a multivariate logistic regression model (backward stepwise regression). A pre-set significance level of *p* < 0.05 and confidence intervals (CI) of 95% were used for all analyses.

## 3. Results

### 3.1. Study Population

**Figure 1:**
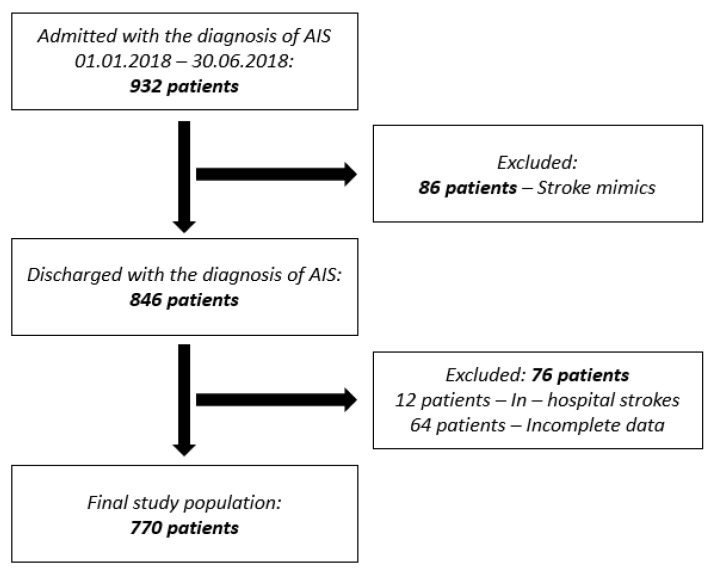
Flowchart of the study population. (*AIS: acute ischemic stroke*).

Between 1 January 2018 and 30 June 2018, 1057 patients were admitted with the diagnosis of acute stroke in the Department of Neurology of the University Emergency Hospital Bucharest, one of the biggest public hospitals in Romania The flowchart of the study population is presented in Figure 1. In total, 932 patients had the admission diagnosis of acute ischemic stroke and 125 patients of acute hemorrhagic stroke; 86 patients with the admission diagnosis of ischemic stroke were excluded from the analysis because they were discharged with another diagnosis. Twelve patients admitted with the diagnosis of acute ischemic stroke were excluded because they had in-hospital strokes. Another 64 patients with acute ischemic strokes were also excluded because data regarding past medical history, prior treatment, moment of stroke onset and other important details needed for the study purpose could not be obtained. Consequently, the study population consisted of 770 patients with acute ischemic stroke.

### 3.2. General Characteristics of the Study Population

The median age of the patients was 73 years (25–75 IQR 64.5–80 years), and 48.8% were males. The median NIHSS score at admission was 6 points (25–75 IQR 3–14), and 66.9% of the patients had anterior circulation strokes. In total, 22.8% of the patients had a priory history of stroke/TIA, 69.9% arterial hypertension, 24.9% diabetes mellitus, 20.7% atrial fibrillation and 16.1% ischemic heart disease. In total, 27.8% of the patients lived in rural areas around Bucharest, and 75.6% of the study population arrived at the hospital by ambulance. The rate of IVT during the study period was 14.1%. Further details about baseline characteristics of the patients and stroke severity at admission are listed in Supplementary Material (Table S1). 

### 3.3. Time Intervals between Stroke Onset and Hospital Arrival

**Figure 2:**
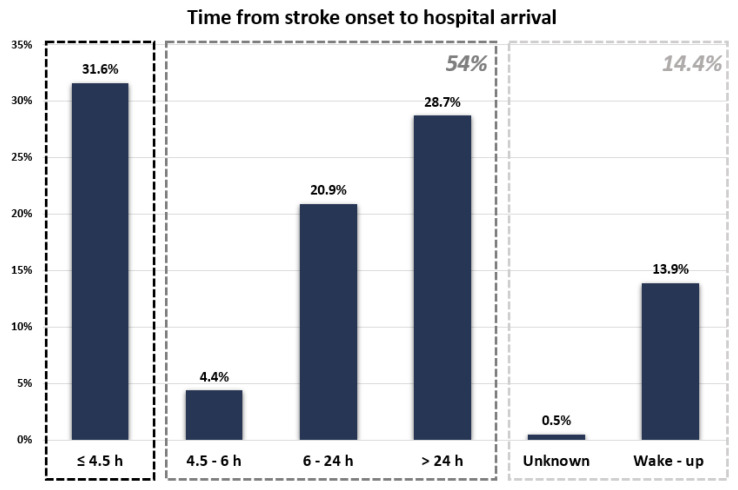
Time between stroke onset and hospital arrival for the study population.

Time intervals between stroke onset and hospital arrival were calculated for all patients included in this study (Figure 1). In total, 243 patients (31.6%) arrived at the hospital within 4.5 h from the moment when they were last seen without symptoms (Figure 2). Another 34 patients (4.4%) arrived at the hospital in time intervals between 4.5 h and 6 h from stroke onset; 161 patients (20.9%) presented to the hospital in the first 24 h after stroke onset but later than 6 h, and 221 patients (28.7%) patients arrived at the hospital after more than 24 h from the moment of stroke onset. For four patients (0.5%), we could establish neither the exact moment of stroke onset nor if the stroke onset was in the preceding 24 h or older than that. These patients had unwitnessed severe strokes and were all living alone; 107 patients (13.9%) had wake-up strokes.

### 3.4. Factors Associated with Late Arrival to Hospital

We defined “early arrival to hospital” as arrival to the hospital within 4.5 h from stroke onset and “late arrival to hospital” as arrival after this time interval. Patients for whom the stroke symptoms were noticed at wake-up were excluded from this analysis if they reached the hospital later than 4.5 h from the moment when they were last time seen well (*n* = 107 patients, 13.9%). Patients for whom the time of stroke onset was unknown at hospital arrival were also excluded from this analysis (four patients, 0.5%). In total, 243 patients (31.5%) arrived at the hospital within 4.5 h from stroke onset and 416 patients (54.1%) after this period. 

The comparative analysis of socio-demographic, clinical and stroke characteristics for patients with early arrival versus late arrival to the hospital is summarized in Table 1. 

Factors associated with a late arrival at the hospital in univariate analysis were: living in rural areas, absence of known history of atrial fibrillation, absence of known history of ischemic heart disease, absence of previous therapy with anticoagulants, transport to hospital by own means, stroke severity at admission, stroke in the vertebrobasilar territory, presence of ataxia and absence of the following stroke signs: hemianopsia, facial palsy, motor weakness, sensory disturbance and speech disturbance. These 13 variables were used to fit a multivariable logistic regression model (backward regression method of entering data). The final model identified the following 6 independent predictors for arrival at hospital after 4.5 h from stroke onset: history of atrial fibrillation, mode of transportation to hospital, initial stroke severity, hemianopsia, sensory disturbances and facial palsy (Table 2). The only factor positively associated with arrival to hospital > 4.5 h from stroke onset was ‘transport to hospital by own means’, which more than doubled the odds of a late arrival (OR 2.2; 95% CI 1.4–3.6). Factors negatively associated with late arrival to the hospital were: known history of atrial fibrillation (OR 0.5; 95% CI 0.3–0.7), initial stroke severity > 15 points on the NIHSS scale (OR 0.6; 95% CI 0.3–0.9), presence of hemianopsia, facial palsy and sensory disturbance. The model as a whole explained between 15.7% (Cox and Snell R^2^) and 21.5% (Nagelkerke R^2^) of the variance in time of hospital arrival and correctly classified 68.6% of the cases (χ^2^ = 110.1, *p* < 0.0001).

### 3.5. Factors Associated with Very Late Arrival to Hospital

We defined “very late arrival to hospital” as arrival to hospital after 24 h from stroke onset. Of the 770 patients included in the study, 545 patients (70.8%) arrived at the hospital within the first 24 h after stroke onset and 221 patients (28.7%) after this time interval. For four patients (0.5%), we could not establish if stroke onset was before or within this time period, and therefore, they were excluded from this analysis.

The comparative analysis of socio-demographic, clinical and stroke characteristics for patients with arrival to the hospital within less than 24 h from stroke onset versus very late arrival to the hospital is summarized in Table 3. 

The factors associated with arrival at hospital after 24 h from stroke onset in univariate analysis were: living alone, living in rural areas, absence of known history of atrial fibrillation, transport to hospital by own means, stroke severity at admission, stroke in the vertebrobasilar territory, presence of ataxia and absence of the following stroke signs: hemianopsia, facial palsy, motor weakness, sensory disturbance, and speech disturbance. 

All these variables were used to fit a multivariate logistic regression model (backward regression method of entering data). Hemianopsia, motor weakness, facial palsy and ataxia were excluded from the final statistical model. Factors increasing the odds of hospital arrival after 24 h from stroke onset in multivariate analysis were living alone (OR 1.7; 95% CI 1.8–2.6) and living in rural areas (OR 1.4; 95% CI 1.01–2.1), and factors decreasing the odds of very late hospital arrival were transport to hospital by ambulance, initial NIHSS score ≥ 16 points, prior diagnosis of atrial fibrillation and speech disturbance (Table 4).

## 4. Discussion

Although more than two decades have passed since rtPA was granted approval for patients with acute ischemic stroke, its use remains restricted to a minority of patients in many regions of the world [11,12]. Consequently, an important body of literature has emerged, presenting a wide variety of factors influencing the likelihood of receiving IVT in case of suffering a stroke. Many of these factors are interrelated, and their individual impact can be heterogeneous, depending on the performance of the different healthcare systems and on the social, cultural, behavioral and economic pattern of the population in which they are studied [13]. 

Numerous studies have assessed the time intervals between stroke onset and hospital arrival, all aiming to identify the most appropriate interventions meant to increase reperfusion treatment rates in various settings [14,15,16]. The comparisons between these studies are somewhat difficult due to multiple methodological variations, especially the definition used for stroke onset (last known without symptoms or first seen with symptoms) and the cut-off value used to exclude pre-hospital time data from patients arriving at the hospital with prolonged delays (e.g., >24 h). 

In a comprehensive review of pre-hospital and in-hospital delay times in acute stroke care performed by Evenson in 2009 [14], the percentage of stroke patients arriving at the hospital in less than 6 h from symptoms onset in studies that did not exclude patients with extreme delay times from the analysis ranged from 17% to 75%. In our study, the percentage of patients who reached the hospital within 6 h from stroke onset was 36%, far lower than in another review of 115 studies, which showed cumulative 6 h percentages of 50% [9]. 

In our study population, transportation to the hospital by own means more than doubled the odds of hospital arrival after 4.5 h from stroke onset, while known history of atrial fibrillation, initial NIHSS score ≤15 points, presence of hemianopsia, facial palsy and sensory disturbance were all factors associated with an early arrival to the hospital.

The many studies analyzing the factors associated with early hospital arrival after stroke performed so far show, almost without exception, that hospital transportation by ambulance is, by far, the factor most frequently associated with a greater likelihood of hospital arrival within the time window for reperfusion therapies [9,14,17,18]. In addition to shortening the pre-hospital delays for stroke patients, use of EMS was also shown to shorten door-to-imaging and door-to-needle times, especially when hospital prenotification is used [19]. The proportion of stroke patients requesting EMS support varies widely from country to country and from region to region. A study performed in 2015 in China [20] showed that only 15.4% of the stroke patients arrived at the hospital by ambulance, while studies performed in other countries showed much higher percentages (Germany 72% [21], England 79% [22], USA 51% [23]). In our study, 75.6% of patients arrived at the hospital by ambulance, and similar to other studies, EMS usage was associated with a higher likelihood of being treated by thrombolysis, as 16.5% of those reaching hospital by ambulance received IVT versus 6.9% of those reaching hospital by private means (*p* = 0.001). 

Several other studies showed that known history of atrial fibrillation increases the likelihood of early hospital arrival, irrespective of stroke severity [24], most probably due to a better knowledge of stroke symptoms which might translate into more appropriate behavior during an acute stroke [16,25]. It can be assumed that information about stroke signs is most probably achieved at the moment of receiving the diagnosis of atrial fibrillation or when oral anticoagulants are prescribed. This finding highlights the opportunity of encouraging primary care physicians to inform all patients with risk factors for stroke about stroke signs and symptoms and the importance of rapid presentation to the hospital. Almost one in three patients in our study population (28.7%) arrived at the hospital more than 24 h after symptom onset. The proportion of patients arriving at the hospital after 24 h from stroke onset in the 23 studies reporting this information, included in the comprehensive review performed by Evenson [14], varied between 11% and 52%, and therefore, we consider the percentage found in our study unexpectedly high, taking into account the habitual geographically small catchment area of the University Emergency Hospital. The analysis of the relationship between different socio-demographic and clinical characteristics of our study population and the moment of hospital arrival, considering the cut-off point of 24 h, showed that factors increasing the odds of hospital arrival after 24 h from stroke onset were living alone and living in rural areas on the city outskirts. These results are in line with other studies from developing countries, which showed consistently longer onset-to-door time intervals for patients from rural areas, outskirts and places with less efficient emergency medical systems and difficult access to medical care [26]. It was also previously reported that patients with mild strokes who either live alone or have a small and close-knit social network are more likely to have late hospital arrival because they selectively disclose symptoms and negotiate a watch-and-wait strategy. This is in contrast to patients with large social networks who are more likely to contact or be surrounded by weakly tied individuals who directly call EMS once symptoms set in [27]. Factors decreasing the odds of arrival at hospital after 24 h from stroke onset in our study were transport to the hospital by ambulance, initial NIHSS score ≥ 16 points, prior diagnosis of atrial fibrillation and speech disturbance. 

These findings could help in the implementation of regional and national stroke awareness campaigns, which should be designed to target not only the population living in urban areas but also the citizens of rural areas. The information included in any future stroke awareness campaign carried out in Romania should firmly convey the message that immediate request of an ambulance, even in the case of minor signs of stroke, is essential for a favorable prognosis. Among European countries, Romania has a leading place in terms of stroke mortality and morbidity, which can be explained not only by the economic status of the country and the poor financing of the health system but also by the traditional unhealthy lifestyle [28]. In 2017, the IVT rate in Romania was 0.8%, and the EVT rate was less than 0.1%, compared to an average of 7.3% IVT rate and 1.9% EVT rate in 44 European Countries [12]. The number of stroke patients treated by IVT and EVT in Romania has steadily increased over the past five years due to the implementation and extension of the Romanian stroke network so that in 2021 the IVT rate reached 5.4% and the EVT rate 0.5%. In 2022, the Romanian stroke network comprises five comprehensive stroke centers that deliver both IVT and EVT and forty-one primary stroke centers that deliver only IVT. A total of 41 counties, along with the municipality of Bucharest, the capital of the country, constitute the official administrative divisions of Romania, and the Romanian stroke network mirrors this frame, with a few exceptions, each county being served by one stroke center. The Romanian Stroke Network is organized exclusively in public hospitals. 

The results of our study suggest that despite the significant extension of the national stroke network, the IVT rate in Romania is unlikely to increase in the near future until the 15% target rate recommended by ESO [29], as just a third of the patients with acute ischemic stroke reach the hospital in less than 4.5 h from stroke onset and almost one-third of the patients reach hospital after more than 24 h from stroke onset. These long time intervals between stroke onset and hospital arrival can be attributed to both the lack of rapid recognition of stroke symptoms by patients and bystanders with inappropriate reactions after stroke onset and to the inability of the Emergency Medical System to act with major priority in case of stroke patients. 

The time from stroke onset or noticing the symptoms to call for help contributes to the largest proportion of the pre-hospital delay time for the majority of patients and is strongly dependent on the general knowledge about stroke and the importance of urgently contacting the EMS. Although most studies proved that awareness of stroke symptoms can be improved by different stroke awareness campaigns, the knowledge seems to be retained for only weeks or months and to not lead to a persistent increase in EMS use [30]. As a result, a stroke awareness campaign, carried out over long periods of time and targeting both potential patients and bystanders, might decrease pre-hospital delays for acute stroke patients in Romania and is urgently needed.

The EMS plays a crucial role for stroke patients not only by diminishing pre-hospital delays but also by facilitating prompt in-hospital evaluation. Correct identification of stroke signs and symptoms in the dispatch center followed by accurate stroke recognition and triage on scene, rapid transportation to facilities able to provide IVT and hospital prenotification are all actions shown to decrease pre-hospital delays and to increase the likelihood of rtPA administration [31], and therefore, all interventions meant to improve each of these actions are crucial in our country for minimizing delays in acute stroke care.

Our study has several limitations. First, it was conducted in a single stroke center, limiting the generalizability of our results for the national level. However, the findings from other regions of the country are expected to be worse, given the lack of stroke awareness campaigns, the absence of dedicated pre-hospital protocols for stroke patients and the larger catchment areas for most of the county hospitals. Second, we defined stroke onset as the moment when a patient was last seen without symptoms and did not register the moment when the patient was first seen with symptoms. Third, we did not analyze bystander-related factors that might have influenced the moment of alerting the EMS services. All these limitations need to be addressed in further studies in order to better understand the underlying factors that preclude stroke patients from receiving IVT in Romania.

## 5. Conclusions

Only one in three ischemic stroke patients presenting to our stroke center reaches the hospital within the 4.5 h accepted time window for IVT, and almost one in three ischemic stroke patients reaches the hospital more than 24 h after stroke onset. These findings highlight the need for urgent measures to improve not only stroke awareness but also pre-hospital protocols in order to provide timely and appropriate care for our stroke patients.

## Figures and Tables

**Table 1:** Baseline characteristics of patients arriving at hospital before and after 4.5 h from stroke onset.

	Arrival ≤ 4.5 h after Stroke Onset (*n* = 243)	Arrival > 4.5 h after Stroke Onset (*n* = 416)	*p* Value
Age, median (25–75 IQR)	73.5 (64–81)	72 (65–80)	0.7
Male sex	111 (45.7%)	206 (49.5%)	0.3
Higher education	39 (16.04)	81 (19.4)	0.4
Place of living and living status			
Living in rural areas *	58 (23.9%)	130 (31.2%)	0.04
Living alone	45 (18.9%)	96 (24.1%)	0.13
Previous history			
Prior stroke/TIA	54 (22.2%)	96 (23.1%)	0.8
Hypertension	171 (70.4%)	286 (68.7%)	0.6
Diabetes mellitus	55 (22.6%)	108 (25.9%)	0.3
Atrial fibrillation (previously diagnosed) *	72 (29.6%)	65 (15.6%)	<0.0001
Ischemic heart disease *	50 (20.6%)	59 (14.2%)	0.03
Current smoking	46 (19.3%)	99 (23.9%)	0.16
Cognitive impairment	27 (11.1%)	49 (11.8%)	0.8
BMI ≥ 30	63 (25.9%)	101 (24.3%)	0.6
Alcohol drinking	43 (17.9%)	84 (20.5%)	0.4
Previous therapy			
Antiplatelets	76 (31.5%)	134 (32.7%)	0.7
Anticoagulants *	44 (18.2%)	44 (10.7%)	0.006
Antihypertensives	149 (61.8%)	231 (56.3%)	0.17
Statins	66 (27.4%)	102 (24.9%)	0.5
Previous level of dependence			0.3
mRS 0–1	166 (70.6%)	280 (70.3%)	
mRS 2–3	38 (16.2%)	78 (19.6%)	
mRS 4–5	31 (13.2%)	40 (10.1%)	
Transport to hospital *			<0.0001 *
By ambulance	208 (85.6%)	282 (67.8%)	
By own means	35 (14.4%)	134 (32.2%)	
NIHSS at admission, median (25–75 IQR) *	10 (5–19)	5 (2–9)	<0.0001 *
Stroke severity *			<0.0001 *
NIHSS ≤ 5	74 (30.4%)	239 (57.4%)	
NIHSS 6–15	87 (35.8%)	120 (28.8%)	
NIHSS ≥ 16	82 (33.7%)	57 (13.7%)	
Stroke territory *			<0.0001 *
Left MCA or ACA	90 (37.2%)	136 (32.7%)	
Right MCA or ACA	97 (40.1%)	114 (27.4%)	
Vertebro-basilar	44 (18.2%)	152 (36.5%)	
Multiple territories	11 (4.5%)	14 (3.4%)	
Stroke signs and symptoms			
Altered level of consciousness	34 (13.9%)	41 (9.8%)	0.13
Hemianopsia *	123 (50.6%)	106 (25.7%)	<0.0001
Facial palsy *	183 (75.3%)	222 (53.6%)	<0.0001
Vertigo	11 (4.5%)	30 (7.2%)	0.2
Motor weakness *	204 (83.9%)	296 (71.3%)	0.0002
Ataxia *	28 (11.6%)	72 (17.3%)	0.04
Sensory disturbance *	97 (39.9%)	88 (21.5%)	<0.0001
Speech disturbance	180 (74.1%)	236 (57%)	<0.0001
Dysarthria *	127 (52.3%)	174 (42.1%)	
Aphasia	53 (21.8%)	62 (14.9%)	

* Result with statistical significance. TIA: transient ischemic attack; mRS: modified Rankin Score; NIHSS: National Institute of Health Stroke Scale; IQR: Interquartile Range; MCA: middle cerebral artery; ACA: anterior cerebral artery.

**Table 2:** Factors associated with hospital arrival after 4.5 h from stroke onset.

	Univariate Analysis		Multivariate Analysis	
	*p* Value	OR (95% CI)	*p* Value	OR (95% CI)
Living in rural areas	0.04	1.4 (1–2.1)	NI	-
Atrial fibrillation *(previously diagnosed)*	<0.0001	0.4 (0.3–0.6)	0.001 *	0.5 (0.3–0.7)
Ischemic heart disease *(previously diagnosed)*	0.03	0.6 (0.4–0.9)	NI	-
Prior treatment with anticoagulants	0.007	0.5 (0.3–0.8)	NI	-
Transport to hospital by own means	<0.0001	2.8 (1.8–4.2)	0.0003 *	2.2 (1.4–3.6)
Stroke severity at admission				
NIHSS < 5	Ref.	Ref.	Ref.	Ref.
NIHSS 5–15	<0.0001	0.4 (0.3–0.6)	NI	NI
NIHSS ≥ 16	<0.0001	0.2 (0.1–0.3)	0.04 *	0.6 (0.3–0.9)
Stroke territory (posterior vs. anterior)	<0.0001	2.6 (1.8–3.8)	0.05	1.5 (0.9–2.3)
Hemianopsia	<0.0001	0.3 (0.2–0.5)	0.03 *	0.6 (0.4–0.9)
Facial palsy	<0.0001	0.4 (0.3–0.5)	0.04 *	0.6 (0.4–0.9)
Motor weakness	0.0002	0.5 (0.3–0.7)	NI	-
Sensory disturbance	<0.0001	0.4 (0.3–0.6)	0.0001*	0.4 (0.3–0.6)
Speech disturbance	<0.0001	0.4 (0.3–0.6)	NI	-
Ataxia	0.04	1.5 (1.01–2.5)	NI	-

* Result with statistical significance. NIHSS: National Intitute of Health Stroke Scale.

**Table 3:** Baseline characteristics of patients arriving at hospital before and after 24 h from stroke onset.

	Arrival ≤ 24 h after Stroke Onset (*n* = 545)	Arrival > 24 h after Stroke Onset (*n* = 221)	*p* Value
Age, median (25–75 IQR)	73 (64–81)	72 (64–78)	0.7
Male sex	274 (50.3%)	101 (45.7%)	0.2
Higher education	106 (19.4%)	38 (17.2%)	0.6
Place of living and living status			
Living in rural areas *	139 (25.5%)	74 (33.5%)	0.02
Living alone *	100 (19.1%)	58 (27.4%)	0.01
Previous history			
Prior stroke/TIA	120 (22.1%)	55 (24.9%)	0.4
Hypertension	383 (70.3%)	153 (69.2%)	0.7
Diabetes mellitus	131 (24.1%)	61 (27.6%)	0.3
Atrial fibrillation (*previously diagnosed*) *	128 (23.5%)	32 (14.5%)	0.005
Ischemic heart disease	94 (17.2%)	30 (13.6%)	0.2
Current smoking	121 (22.6%)	48 (21.7)	0.8
Cognitive impairment	57 (10.4%)	29 (13.1%)	0.3
BMI ≥ 30	143 (26.2%)	54 (24.4%)	0.6
Alcohol drinking	108 (20.2%)	41 (18.8%)	0.6
Previous therapy			
Antiplatelets	165 (30.6%)	70 (32.1%)	0.7
Anticoagulants	78 (14.5%)	27 (12.4%)	0.4
Antihypertensives	324 (60.1%)	117 (53.6%)	0.1
Statins	139 (25.8%)	56 (25.7%)	0.9
Previous level of dependence			0.7
mRS 0–1	357 (67.9%)	149 (70.9%)	
mRS 2–3	104 (19.8%)	39 (18.6%)	
mRS 4–5	65 (12.4%)	22 (10.5%)	
Transport to hospital *			<0.0001
By ambulance	445 (81.6%)	133 (60.2%)	
By own means	100 (18.4%)	88 (39.8%)	
NIHSS score at admission, median (25–75 IQR) *	7 (4–16)	4 (2–8)	<0.0001
Stroke severity *			<0.0001
NIHSS ≤ 5	215 (39.4%)	137 (61.9%)	
NIHSS 6–15	182 (33.4%)	61 (27.6%)	
NIHSS ≥ 16	148 (27.2%)	23 (10.4%)	
Stroke territory *			0.0001
Left MCA or ACA	201 (36.9%)	68 (30.8%)	
Right MCA or ACA	188 (34.5%)	55 (24.9%)	
Vertebro-basilar	134 (24.6%)	91 (41.2%)	
Multiple territories	21 (3.8%)	7 (3.2%)	
Stroke signs and symptoms			
Altered level of consciousness	72 (13.2%)	20 (9.1%)	0.1
Hemianopsia *	223 (40.9%)	48 (21.9%)	<0.0001
Facial palsy *	375 (68.9%)	106 (48.2%)	<0.0001
Vertigo	33 (6.1%)	16 (7.3%)	0.5
Motor weakness *	436 (80%)	150 (68.2%)	0.0004
Ataxia *	69 (12.7%)	42 (19%)	0.02
Sensory disturbance *	169 (31.12%)	50 (22.7%)	0.02
Speech disturbance	393 (72.2%)	106 (48.2%)	<0.0001
Dysarthria *	292 (53.7%)	78 (35.5%)	
Aphasia	101 (18.5%)	28 (12.7%)	

* Result with statistical significance. IQR: interquartile range; TIA: transient ischemic attack; mRS: modified Rankin Score; NIHSS: National Institute of Health Stroke Scale; MCA: middle cerebral artery; ACA: anterior cerebral artery.

**Table 4:** Factors associated with hospital arrival after 24 h from stroke onset.

	Univariate Analysis		Multivariate Analysis	
	*p* Value	OR (95% CI)	*p* Value	OR (95% CI)
Living alone	0.01	1.6 (1.1–2.3)	0.001 *	1.7 (1.8–2.6)
Living in rural area	0.02	1.5 (1.1–2.1)	0.04 *	1.4 (1.01–2.1)
Atrial fibrillation	0.005	0.5 (0.3–0.8)	0.03 *	0.6 (0.4–0.9)
Transport to hospital by ambulance	<0.0001	0.3 (0.2–0.4)	<0.0001 *	0.4 (0.3–0.6)
Stroke severity at admission				
NIHSS ≤ 5	Ref.	Ref.	Ref.	Ref.
NIHSS 6–15	0.001	1.9 (1.2–2.8)	NI	NI
NIHSS ≥ 16	<0.0001	4.1 (2.3–6.7)	0.03 *	0.5 (0.3–0.9)
Stroke territory (posterior vs. anterior)	<0.0001	2.1 (1.5–2.9)	NS	NS
Hemianopsia	<0.0001	0.4 (0.2–0.5)	NI	NI
Facial palsy	<0.0001	0.4 (0.3–0.6)	NI	NI
Motor weakness	0.0005	0.5 (0.3–0.7)	NI	NI
Sensory disturbance	0.02	0.6 (0.4–0.9)	NS	NS
Speech disturbance	<0.0001	0.3 (0.2–0.4)	0.002 *	0.4 (0.3–0.7)
Ataxia	0.02	0.6 (0.4–0.9)	NI	NI

* Result with statistical significance. NIHSS: National Institute of Health Stroke Scale.

## Data Availability

Data used in this study may be provided by the corresponding author upon reasonable requests.

## Supplementary Materials

The following supporting information can be downloaded at: https://www.mdpi.com/article/10.3390/medicina58081003/s1, Table S1: Baseline characteristics of the study population, Questionnaire regarding pre-hospital delay for patients with acute ischemic stroke.
