# Supplementary material for: Pre-Hospital Delay in Acute Ischemic Stroke Care: Current Findings and Future Perspectives in a Tertiary Stroke Center from Romania—A Cross-Sectional Study
Source: Medicina (Kaunas). 2022 Jul 27;58(8):1003. doi: 10.3390/medicina58081003 (PMC9415394; doi:10.3390/medicina58081003)
Supplement: Supplementary file 1 [file medicina-58-01003-s001.zip › medicina-1772738-supplementary.pdf]

Table S1. Baseline characteristics of the study population.

	n **(%)***
Number of patients	770
Age (years), median (25 – 75 IQR)	73 (64.5 – 80)
Male sex	376 (48.8%)
Previous history	
Previous stroke/TIA	176 (22.8%)
Hypertension	538 (69.9%)
Diabetes mellitus	192 (24.9%)
Atrial fibrillation	160 (20.7%)
Ischemic heart disease	124 (16.1%)
Active smoking/ former smoker	169 (22.2%)
Cognitive impairment	86 (11.2%)
BMI ≥ 30	198 (25.7%)
Previous therapy	
Antiplatelets	236 (31.1%)
Anticoagulants	105 (13.8%)
Antihypertensives	443 (58.2%)
Statins	196 (25.7%)
Origin	
Home	678 (88.2%)
Other hospital/medical institution/nursing home	71 (9.2%)
Public place	21 (2.6%)
Place of living	
Urban areas	556 (72.2%)
Rural areas	214 (27.8%)
Education	
Primary/secondary education	626 (81.3%)
Higher education	144 (18.7%)
Living status	
Lives alone	167 (21.7%)
Lives with family	603 (78.3%)
Previous level of dependence	
mRS 0-1	530 (68.9%)
mRS 2-3	149 (19.3%)
mRS 4-5	91 (11.8%)
Transport to hospital	
By ambulance	582 (75.6%)
By own means	188 (24.4%)
Stroke severity	
NIHSS at admission, median (25-75 IQR)	6 (3-14)
Stroke territory	
Left MCA or ACA	268 (35.1%)
Right MCA or ACA	243 (31.8%)
Vertebro-basilar	225 (29.4%)
Multiple territories	28 (3.7%)
Treatment by IVT	109 (14.1%)
** unless otherwise specified*	

Questionnaire regarding pre-hospital delay for patients with acute ischemic stroke

1. Stroke symptoms were first noticed at wake – up? YES/ NO

2. Were there any witnesses at the time of stroke onset? YES/ NO

3. Date and time when the patient was last seen without symptoms:
   Date _ _/_ _ _/_ _ _ _ (DD/MM/YEAR)
   Time _ _: _ _

4. *For patients with wake-up strokes*
   Date and time when the patient went to sleep:
   Date _ _/_ _ _/_ _ _ _ (DD/MM/YEAR)
   Time _ _: _ _

   Date and time of awakening:
   Date _ _/_ _ _/_ _ _ _ (DD/MM/YEAR)
   Time _ _: _ _

5. Date and time of first call to Ambulance:
   Date _ _/_ _ _/_ _ _ _ (DD/MM/YEAR)
   Time _ _: _ _

6. Date and time of hospital arrival:
   Date _ _/_ _ _/_ _ _ _ (DD/MM/YEAR)
   Time _ _: _ _
